# Association of CaMK2A and MeCP2 signaling pathways with cognitive ability in adolescents

**DOI:** 10.1186/s13041-021-00858-8

**Published:** 2021-10-04

**Authors:** Li-Ching Lee, Ming-Tsan Su, Hsing-Ying Huang, Ying-Chun Cho, Ting-Kuang Yeh, Chun-Yen Chang

**Affiliations:** 1 Science Education Center and Graduate Institute of Science Education, National Taiwan Normal University, No. 88, Sec. 4, Ting-Chou Rd., Taipei, 11677 Taiwan, Republic of China; 2 Department of Life Science, National Taiwan Normal University, Taipei, Taiwan; 3 Institute of Marine Environment Science and Technology, National Taiwan Normal University, Taipei, Taiwan; 4 Department of Earth Science, National Taiwan Normal University, Taipei, Taiwan

**Keywords:** Glutamatergic signaling pathway, Single-nucleotide variant (SNV), Methyl-CpG binding protein 2 (MeCP2), Calcium/calmodulin-dependent protein kinase IIα (CaMK2A), Cognitive function

## Abstract

The glutamatergic signaling pathway is involved in molecular learning and human cognitive ability. Specific single variants (SNVs, formerly *single-nucleotide polymorphisms*) in the genes encoding N-methyl-d-aspartate receptor subunits have been associated with neuropsychiatric disorders by altering glutamate transmission. However, these variants associated with cognition and mental activity have rarely been explored in healthy adolescents. In this study, we screened for SNVs in the glutamatergic signaling pathway to identify genetic variants associated with cognitive ability. We found that SNVs in the subunits of ionotropic glutamate receptors, including *GRIA1*, *GRIN1*, *GRIN2B*, *GRIN2C*, *GRIN3A*, *GRIN3B*, and calcium/calmodulin-dependent protein kinase IIα (*CaMK2A*) are associated with cognitive function. Plasma CaMK2A level was correlated positively with the cognitive ability of Taiwanese senior high school students. We demonstrated that elevating CaMK2A increased its autophosphorylation at T286 and increased the expression of its downstream targets, including GluA1 and phosphor- GluA1 in vivo. Additionally, methyl-CpG binding protein 2 (MeCP2), a downstream target of CaMK2A, was found to activate the expression of CaMK2A, suggesting that MeCP2 and CaMK2A can form a positive feedback loop. In summary, two members of the glutamatergic signaling pathway, CaMK2A and MeCP2, are implicated in the cognitive ability of adolescents; thus, altering the expression of CaMK2A may affect cognitive ability in youth.

## Introduction

N-methyl-d-aspartate receptors (NMDARs) are ionotropic glutamate receptors crucial for neuronal communication, which plays a central role in learning, memory, and synaptic development. NMDARs form tetrameric complexes that consist of two glutamate ionotropic receptor NMDA type subunit 1 (GluN1) subunits and two GluN2 or GluN3 subunits [1–3]. Although NMDARs are widely expressed throughout the central nervous system (CNS), their number, localization, and subunit composition are strictly regulated and differ by cell and synapse. All NMDAR subunits contain modular domains that are responsible for controlling distinct functions. All of the ionotropic glutamate receptor subunits, including the seven GluNs, share a common membrane topology; however, the GluN1 isoforms and GluN2 subunits exhibit developmental and regional variations [4–8]. Approximately 2 decades ago, the GluN3A and GluN3B subunits were the last NMDAR subunits to be cloned [9, 10]. GluN3A expression is low before birth, peaks during early postnatal life, and decreases to low levels in adulthood [11]. By contrast, GluN3B subunit expression is low in early life but increases progressively through adulthood [12].

Synaptic proteins play a crucial role in synaptic activity and dendritic spine morphogenesis, and variations in synaptic proteins lead to cognitive deficits [13]. Because cognitive ability directly affects intellectual capacity, these proteins are associated with youth academic achievement [14]. Of all the genes involved in cognitive function, members of the glutamatergic signaling pathway are the most interesting [15]. Glutamate is a major excitatory neurotransmitter involved in learning and memory, long-term potentiation (LTP), and synaptic plasticity [16]. In neurons, glutamate binds to and activates ionotropic receptors (e.g., NMDA) and α-amino-3-hydroxy-5-methyl-4-isoxazolepropionic acid receptors (AMPARs) and mediates Ca^2+^ transport, thus activating intracellular signaling cascades to alter synaptic efficacy and induce LTP [17].

A signaling pathway activated by ionotropic receptor-mediated Ca^2+^ influx involves Ca^2+^/calmodulin (CaM)-dependent protein kinase II (CaMKII), a serine/threonine kinase enriched at excitatory synapses and postsynaptic densities [18]. Upon binding with Ca^2+^/CaM, CaMKII phosphorylates numerous substrates responsible for LTP, including voltage- and ligand-gated Ca^2+^ channels, cAMP-response element-binding protein, extracellular signal-regulated kinase (ERK), and voltage-gated sodium channels [19]. After initial activation by Ca^2+^-bound CaM, CaMKII autophosphorylates at Thr286/287, thus enhancing its binding affinity for Ca^2+^/CaM and inducing Ca^2+^/CaM-independent autonomous phosphorylation [20]. This autonomous kinase activity of CaMKII has been hypothesized as providing biochemical memory storage for LTP [18, 21]. Blockade of the autophosphorylation of CaMK2A (Thr 286), a forebrain-enriched CaMKII isoform, in knock-in mice expressing phospho-dead CaMKII^*T286A*^ variant proteins impaired LTP, sLTP, and spatial learning and memory [22].

Several recent studies have provided novel insights into the synaptic mechanisms of pathological pathways and have demonstrated that de novo variations in CaMK2A disrupt the function of synaptic proteins [23]. For example, a Glu831 to Val (CaMK2A^*E831V*^) variation in the CaMK2A catalytic domain reduces both CaMK2A substrate phosphorylation and regulatory autophosphorylation. Additionally, CaMK2A^*E831V*^ may inhibit the phosphorylation of CaMK2A in a dominant-negative manner [24]. Loss of function from *CaMK2A* variation (CaMK2A^*H477Y*^) causes growth delay and seizures in humans [25]. Related studies of two de novo variants in *CaMK2A* and *CaMK2B* have demonstrated that Thr286/Thr287 plays a pivotal role in neuronal plasticity [23, 26]. Activated CaMK2A targets dendritic spines and postsynaptic density through interactions with various CaMKII-associated proteins, including GluN2B NMDAR subunits [27].

CaMKII also functions in the nucleus through nuclear Ca^2+^ signaling. One principal substrate of CaMKII is methyl-CpG binding protein 2 (MeCP2). During neuronal activity and the subsequent Ca^2+^ influx, CaMKII phosphorylates MeCP2 at S421 [28, 29]. This neuronal activity-induced phosphorylation is essential for numerous neuronal functions and neurodevelopment [30]. MeCP2 phosphorylation at various sites regulates numerous target genes, including brain-derived neurotrophic factor (*BDNF*), ras-related GTP-binding protein 3d (*Rab3d*), vesicle-associated membrane protein 3(*Vamp3*), and cell adhesion molecule (*CADM3*). The neuronal activity-induced phosphorylation of MeCP2 might function as a molecular switch regulating the dynamic expression of neuronal genes [31].

MeCP2 is an X-linked global transcription regulator that binds to methylated sites in DNA, and its dysfunction is implicated in Rett syndrome (RTT) and MeCP2 duplication syndrome (MDS). Well-defined mouse models of both syndromes have resulted in learning and memory impairment [32–34]. Apart from the neuropathological lesions caused by MeCP2-related disorders, the mechanisms through which MeCP2 affects learning and cognitive ability (e.g., intelligence quotient) remain unknown. Research on the underlying pathophysiological mechanisms of RTT and MDS advanced our understanding of MeCP2 functions in the nervous system [35]. After screening adolescents for genetic and epigenetic factors associated with cognitive ability, we previously reported that multiple epigenetic biomarkers regulating MeCP2 homeostasis are associated with academic performance [36]. Because components of glutamatergic signaling, including, CaMK2A, NMDARs, and AMPARs, are essential for appropriate synaptic development and plasticity, and their disruption leads to cognitive deficits, we speculated that subtle and chronic alterations in these genes might affect the cognitive ability of students.

Signaling modulators, including CaMK2A and MeCP2, impair cognitive function in many neuropsychiatric disorders [37–39]. Although the molecular mechanisms underlying disease progression have been determined, the roles of these genes in the cognitive function of healthy adolescents have not been investigated. To identify genetic and epigenetic factors associated with adolescent cognitive ability, we demonstrated that multiple epigenetic biomarkers regulate MeCP2 homeostasis and are associated with academic performance [36]. In this study, we investigated genetic factors associated with youth cognitive ability. We discovered that single-nucleotide variants (SNVs) in *CaMK2A* and the subunits of ionotropic glutamate receptors, including *GRIN1*, *GRIN2B*, *GRIN2C*, *GRIN3A*, *GRIN3B*, *GRIA1*, and *GRID1*, are associated with student cognitive function. Furthermore, we discovered that CaMK2A levels were elevated in the peripheral blood samples of senior high school students with superior reasoning skills. Elevated CaMK2A increased the pCaMK2A and GluA1 in cells. Moreover, a downstream target of CaMK2A, MeCP2 could increase the expression of CaMK2A, suggesting the CaMK2A and MeCP2 could form an autoregulatory positive feedback signal transduction loop. We believed that alteration in the expression of CaMK2A might affect the cognitive ability of adolescents through altering the expression of the components of the glutamatergic signaling pathway.

## Materials and methods

### Participants

A total of 832 students (269 males and 563 females, aged 16.3 ± 0.5 years) from three senior high schools (one each in Southern, Central, and Northern Taiwan) were recruited. This study was approved by the Institutional Review Board of National Taiwan University Hospital (Research Ethics identifier: NCT00713570). The volunteers and their parents were explicitly informed, and written consent was obtained.

### Genetic screening, variation analysis, and bioinformatics

DNA samples from 20 healthy participants were first genotyped in a pilot study. SNVs with a minor allele frequency of > 5% were selected. The selected SNVs were then genotyped for all participants. Genotyping was conducted through DNA sequencing of relevant polymerase chain reaction (PCR) products using Prism_ BigDye Terminator v3.1 Cycle Sequencing Ready Reaction Kits and a Prism_ 3730 Genetic Analyzer (Applied Biosystems, Foster City, CA, USA) following manufacturer instructions.

### Cognitive ability assessments

Cognitive ability was assessed with the Multiple Aptitude Test Battery (MAT) [40, 41]. A Chinese version of the MAT was created from the Differential Aptitude Test [41]. The revised MAT was standardized for measuring the cognitive ability of Taiwanese adolescents [42] and comprises eight subtests: verbal reasoning, numerical ability, mechanical reasoning, perceptual speed and accuracy, spatial relations, abstract reasoning, verbal comprehension, and grammar and language. The test contains 496 items and requires approximately 80 min to complete; consistency reliability ranges from 0.5 to 0.9.

### Blood samples and enzyme-linked immunosorbent assay

Blood samples were collected from the participants by using ethylenediaminetetraacetic acid (EDTA) as an anticoagulant and placed on ice. The samples were then centrifuged at 4 °C for 10 min at 3000*g*. Plasma was collected and stored at − 80 °C until use. Platelets were removed through centrifugation for 10 min at 10000*g*. CaMK2A levels were measured using a quantitative enzyme-linked immunosorbent assay (ELISA) kit following the manufacturer’s instructions (LifeSpan BioSciences, Inc. WA, USA).

### CaMK2A cDNA constructs and cloning

**Fig. 1:**
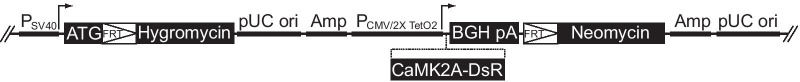
Schematic outline (not to scale) of the Flp-In™ stable cell lines with inducible expression of CaMK2A–DsR fused gene. The CaMK2A–DsR expression cassette is integrated into a distinct Flp recombination target site and is under the control of the Tet repressor. CaMK2A expression is induced by tetracycline. P: promoter; SV40: simian virus 40; CMV: cytomegalovirus; lacZ: β-galactosidase ORF, Amp: ampicillin ORF, DsR: DsRed ORF, pUC ori: Replication origin of plasmid pUC, TetO2: Tetracycline operator 2, and BGH pA: Bovine growth hormone polyadenylation signal

CaMK2A mRNA (cDNA clone Mammalian Gene Collection (MGC): 95) was purchased and reverse transcribed to generate CaMK2A cDNA (Bioresource Collection and Research Center, Taiwan). The CaMK2A cDNA was digested with NotI and ApaI restriction endonucleases and cloned with N-terminal-tagged enhanced green fluorescent protein or C-terminal-tagged DsR into a pcDNA/Flp recombination Target (FRT)/TO cloning vector (Thermo Fisher Scientific Co. MA USA) (Fig. 1).

### Cell culture and transfection

The Flp-In T-REx system was used to stably induce the expression of target genes in the HEK293 and SH-SY5Y cell lines (Invitrogen, Carlsbad, CA, USA). HEK293-derived Flp-In host cells were purchased (Flp-In T-REx Cell Line, Invitrogen). The generation of the SH-SY5Y-derived Flp-In host cells was described previously [43]. In brief, the two cell lines were cotransfected with a pOG44 plasmid (constitutively expressing Flp recombinase) and a pcDNA5/FRT/TO-CaMK2A or pcDNA5/FRT/TO-MeCP2 plasmid following the supplier’s instructions. These stable cell lines were cultured in a medium containing 5 mg/mL blasticidin and 100 mg/mL hygromycin. Doxycycline (1 mg/mL) was added for 2–6 days to induce CaMK2A and MeCP2 expression.

### Western blotting

Total soluble protein was extracted from the HEK293-derived and SH-SY5Y-derived cells treated with 3 μM CaM (Bovine brain Millipore Sigma-Aldrich, MO, USA) at designated time points (0, 2, 4, and 6 days) using a buffer containing 50 mM Tris–HCl, 150 mM NaCl, 1 mM EDTA, 1 mM ethylene glycol-bis(β-aminoethyl ether)-N, N, N′, N′-tetraacetic acid, 0.1% SDS, 0.5% sodium deoxycholate, 1% Triton X-100, and a protease inhibitor cocktail (Life Technologies [Thermo Fisher Scientific], Carlsbad, CA, USA). After sonication and centrifugation at 15000*g* for 10 min at 4 °C, protein concentration was determined (Bio-Rad Protein Assay, Hercules, CA, USA) with bovine serum albumin as the standard. Soluble proteins (25 μg) were separated through 12% sodium dodecyl sulfate–polyacrylamide gel electrophoresis (SDS-PAGE). The proteins were electroblotted onto nitrocellulose membranes, which were blocked with 10% nonfat milk and probed with primary antibodies. The antibodies and dilutions used were as follows: anti-MeCP2 (1:2000; GeneTex), anti-pMeCP2 (phospho-S80; 1:1000; GeneTex), anti-CaMK2A (1:1000; GeneTex), anti-pCaMK2A (phospho-T286; 1:1000; GeneTex), anti-BDNF (1:2000; GeneTex), anti-GluA1 (1:2000; Abcam, Cambridge, UK), anti-GluA1 (phospho-S831; 1:1000; Abcam), and anti-H3.3B (1:2000; GeneTex) antibodies. Immune complexes were detected using horseradish peroxidase-conjugated goat anti-mouse, goat anti-rabbit (Jackson Immuno Research, West Grove, PA, USA) IgG (1:10,000), and a chemiluminescent substrate (Millipore, Burlington, MA, USA).

### Chromatin immunoprecipitation

The SH-SY5Y-derived cells were treated with formaldehyde (1% final concentration) for 10 min at 37 °C and quenched with 0.125 M glycine for 5 min at 25 °C. After being washed twice with PBS, the samples were homogenized in lysis buffer (1% SDS, 10 mM EDTA, 50 mM Tris; pH 8.1) containing a protease inhibitor (Thermo Fisher Scientific MA, USA). Chromatin was sheared using a Bioruptor sonicator for 35 30-s-ON/30-s-OFF cycles in a 4 °C water bath. For immunoprecipitation, 150 μL of chromatin was diluted at a ratio of 1:10 in chromatin immunoprecipitation (ChIP) dilution buffer (0.01% SDS, 1.1% Triton X-100, 1.2 mM EDTA, 16.7 mM Tris–HCl, 167 mM NaCl; pH 8.1). Approximately 7% of the diluted sample was used as input control. Protein A/G Magnetic Beads (Merck Millipore, MA, USA) were incubated with anti-MeCP2 antibodies (GeneTex) or rabbit IgG overnight at 4℃ and constantly rotated in a blocking solution (0.5% BSA in PBS). After washing and resuspension of the antibody–bead conjugates, the chromatin samples were added to the antibody-bead conjugates and incubated for 16 h and rotated constantly at 4℃. After immunoprecipitation, the beads were washed six times (5 min each) with buffer (50 mM HEPES–KOH, 500 mM LiCl, 1 mM EDTA, 1% NP-40, 0.7% Na-deoxycholate; pH 7.6). and washed with TE buffer (10 mM, 1 mM EDTA; pH 8.1). DNA-proteins complexes were eluted with a 200-μL elution buffer (50 mM Tris–HCl, 1 mM EDTA, 1% SDS; pH 8.1). The eluted samples were incubated at 65 °C for 4 h, and the input DNA was diluted in elution buffer (to 200 μL) and processed for cross-link reversal. The samples were then digested sequentially in 0.2 μg/mL RNase A for 2 h at 37 °C and 20 μg of proteinase K for 30 min at 55 °C. The DNA was extracted using the phenol/chloroform/isoamyl alcohol method and subjected to quantitative PCR using the ViiA 7 real-time PCR system (Thermo Fisher Scientific MA, USA) with primer pairs for *GRINI*, *GRIA1*, and *GRID1*.

### Statistical analysis

The associations of genotypes with the participants' cognition (MAT scores) were assessed through analysis of variance (One-way ANOVA). Univariate ANOVA was performed to compare MAT subtest scores among the three genotype groups for each SNV. Significance was set at *p* < 0.05. Bonferroni correction was used for multigroup comparisons. Post hoc Scheffe’s F testing was performed because of its high statistical power [44]. For each SNV, the participants were assigned to one of three groups based on their genotype, and deviations from the Hardy–Weinberg equilibrium were tested using a chi-squared test. Gene–gene interactions were assessed using analysis of covariance. The ANOVAs and inferential statistical analyses were conducted in SPSS version 23.0.

## Results

### Gene screening, variation analysis, and bioinformatics

Our previous study discovered that genetic variants in glutamatergic signaling components were associated with the emotions and social behaviors of adolescents [45]. In the present study, 832 10th-grade (269 male; 563 female) volunteers were recruited for the study. DNA samples from 20 healthy participants were genotyped in a pilot study. From the set of candidate genes associated with NMDARs, 174 SNVs were identified using information available in the Entrez Gene (http://www.ncbi.nlm.nih.gov/gene), HapMap (http://www.hapmap.org), and Ensembl (http://www.ensembl.org/Homo_sapiens) databases. We found that SNVs in genes encoding NMDAR subunits were associated with academic performance and cognitive ability (Table 1). The observed genotype distribution for each SNV was consistent with the Hardy–Weinberg equilibrium. Furthermore, the genotype frequencies for each SNV in the study population were consistent with the HapMap–HCB population study results reported by the International HapMap Project.

**Table 1:** Demographics

	Male	Female	*p*
Age (yrs)	16.8 ± 0.32^*^	16.8 ± 0.30	
Academic performance			
BCT	245.9 ± 19.69	235.8 ± 20.61	< 0.01
Cognitive abilities			
Verbal reasoning	21.5 ± 5.31	21.9 ± 5.16	
Numerical ability	11.1 ± 3.56	10.90 ± 3.37	
Mechanical reasoning	14.3 ± 3.91	13.3 ± 3.46	< 0.01
Space relations	17.1 ± 4.49	15.4 ± 4.49	< 0.01
Abstract reasoning	20.2 ± 4.81	19.1 ± 4.95	< 0.01
Verbal comprehension	21.5 ± 6.14	22.2 ± 5.91	< 0.05
Grammar and language usage	17.9 ± 5.04	18.5 ± 5.00	< 0.05
Perceptual speed and accuracy	67.2 ± 21.33	68.1 ± 20.19	

^*^Mean (± standard deviation); BCT, basic competency test

In total, 26 SNVs with a minor allele frequency greater than 5% in the pilot study were identified as candidates and used for further genotyping experiments for all 832 volunteers. We discovered that 13 SNVs were significantly associated with cognitive ability (Table 3). The participants’ background (age, years of education, academic performance; Table 1) and MAT subtest (verbal reasoning, numerical ability, mechanical reasoning, perceptual speed and accuracy, spatial relations, abstract reasoning, verbal comprehension, and grammar and language usage) scores for the genotype groups for each SNV were analyzed through ANOVA (Tables 2 and 3).

**Table 2:** Genotype distributions and chromosome locations of SNVs

Gene	SNP ID	Allele/genotype	Subjects	Chromosome region	Genotype frequency
*GRIN1*				9q34.3	
	rs4880213	CC/CT/TT	124/368/340		0.15/0.44/0.41
	rs11146020	CC/CG/GG	565/239/28		0.68/0.29/0.03
*GRIN2B*				12p12	
	rs3764028	CC/CG/GG	250/396/186		0.30/0.48/0.22
	rs1806201	AA/AG/GG	223/438/171		0.27/0.53/0.20
	rs1805247	AA/AG/GG	608/204/20		0.73/0.25/0.02
	rs1805502	AA/AG/GG	608/203/20		0.73/0.24/0.03
	rs7301328	AA/AC/CC	148/397/287		0.18/0.48/0.34
*GRIN2C*				17q25	
	rs3744215	AA/AC/CC	143/423/266		0.17/0.51/0.32
*GRIN3A*				9q34.1	
	rs10989591	CC/CT/TT	738/90/4		0.89/0.11/0.01
	rs10989589	CC/CT/TT	527/265/40		0.63/0.32/0.05
	rs3739722	CC/CT/TT	508/287/37		0.61/0.35/0.04
	rs62000403	TT/AT/AA	708/115/9		0.85/0.14/0.01
*GRIN3B*				19p13.3	
	rs2240154	CC/CT/TT	254/405/173		0.31/0.49/0.21
	rs35592366	CC/AC/AA	627/191/14		0.75/0.23/0.02
	rs55646937	GG/AG/AA	562/231/39		0.58/0.28/0.05
	rs12978900	TT/CT/TT	697/133/2		0.84/0.16/0.01
	rs4807399	CC/CT/TT	707/119/6		0.86/0.14/0.01
	rs2240157	CC/CT/TT	207/413/212		0.25/0.50/0.26
	rs10666583	MM/Mm/mm	730/98/4		0.88/0.12/0.01
	rs2240158	CC/CT/TT	597/214/21		0.72/0.26/0.03
	rs2285906	GG/AG/AA	626/183/23		0.75/0.22/0.03
	rs10417824	TT/AT/AA	362/355/115		0.23/0.22/0.08
	rs10401454	CC/CG/GG	622/163/47		0.75/0.23/0.02
*GRIA1*				5q33.2	
	rs548294	CC/CT/TT	374/372/86		0.45/0.45/0.10
*GRID1*				10q23.1-2	
	rs3814614	GG/AG/AA	522/266/42		0.63/0.32/0.05
*CaMK2A*				5q32	
	rs2241694	GG/AG/AA	580/235/16		0.70/0.28/0.02

**Table 3:** SNVs associated with cognitive ability and synaptic plasticity of glutamatergic neurons

Gene	SNP	Cognitive abilities	Verbal reasoning	Numerical ability	Mechanical reasoning	Space relations	Abstract reasoning	Verbal comprehension	Grammar and language	Perceptual speed and accuracy
*GRIN1*	rs4880213									F = 4.090 P = 0.018
*GRIN2B*	rs1805502						F = 3.827 P = 0.022		F = 2.948 P = 0.053	
*GRIN2C*	rs3744215				F = 4.340 P = 0.013					
*GRIN3A*	rs10989589							F = 4.081 P = 0.017		
	rs3739722						F = 3.420 P = 0.033			
*GRIN3B*	rs2240154						F = 4.423 P = 0.012			
	rs4807399								F = 3.755 P = 0.024	
	rs2240157				F = 3.697 P = 0.025		F = 3.360 P = 0.035			
	rs2285906		F = 4.103 P = 0.017		F = 4.034 P = 0.018		F = 3.543 P = 0.030			
	rs10417824			F = 3.064 P = 0.047			F = 4.454 P = 0.012			F = 4.056 P = 0.018
	rs10401454		F = 5.412 P = 0.005			F = 3.158 P = 0.043	F = 4.883 P = 0.008	F = 5.123 P = 0.006	F = 5.832 P = 0.003	F = 3.437 P = 0.033
GRIA1	rs548294				F = 4.000 P = 0.019					
*CaMK2A*	rs2241694									F = 3.248 P = 0.041

F, F ratio = Between estimate/within estimate = (SSB/dfb)/(SSW/dfw) SSB, Sum of squares between groups, SSW: Sum of squares within groups; dfb, Between groups degrees of freedom; dfw, Within groups degrees of freedom

P, *p* < 0.05

NMDARs are glutamate-gated cation channels that are expressed throughout the brain and play an essential role in physiological and pathological processes in the CNS. The spatiotemporal expression of the diverse subunits imparts distinct channel kinetics, permeation, blockage by divalent cations, and sensitivity to endogenous modulators. The *GRIN1* rs4880213 variant was significantly associated with perceptual speed and accuracy (*p* = 0.018). The *GRIN2B* rs1805502 variant was significantly associated with abstract reasoning and grammar and language usage (*p* = 0.022 and *p* = 0.053), and the *GRIN2C* rs3744215 variant was significantly associated with mechanical reasoning (*p* = 0.013). Two SNVs in *GRIN3A* (rs10989589 and rs3739722) were significantly associated with verbal comprehension and abstract reasoning (*p* = 0.017 *p* = 0.033). The other six SNVs in *GRIN3B* were significantly associated with the eight MAT subtest scores as follows. *GRIN3B* rs2240154 was significantly associated with abstract reasoning (*p* = 0.012). *GRIN3B* rs4807399was significantly associated with abstract reasoning and grammar and language usage (*p* = 0.035 and *p* = 0.024). *GRIN3B* rs2240157 was significantly associated with abstract reasoning (*p* = 0.025). *GRIN3B* rs2285906 was significantly associated with mechanical reasoning, abstract reasoning, and perceptual speed and accuracy (*p* = 0.018, *p* = 0.03, and *p* = 0.018). *GRIN3B* rs10417824 was significantly associated with abstract reasoning and perceptual speed and accuracy (*p* = 0.012 and *p* = 0.033). *GRIN3B* rs10401454 was significantly associated with scores on five of the eight MAT subtests, namely the verbal reasoning, spatial relations, abstract reasoning, verbal comprehension, and grammar and language usage (*p* = 0.005, *p* = 0.043, *p* = 0.008, *p* = 0.006, and *p* = 0.003, respectively) scores, as listed in Table 3.

### Plasma CaMK2A level was positively correlated with cognitive abilities

To assess cognitive ability using the MAT, only students without physical or mental disorders were included in our study. The *GRIA1* rs548294 and *CaMK2A* (rs2241694) SNVs were highly associated with cognitive ability, prompting us to study how they affect gene function and how these genes affect cognitive ability. The *CaMK2A* (rs2241694) variant was significantly associated with perceptual speed and accuracy (*p* = 0.041). To address this, 120 students were selected for the study based on their MAT scores. Sixty students with MAT scores higher than 80% were included in a high–cognitive ability group, and 60 students with MAT scores lower than 12% were considered a low–cognitive ability group. Plasma CaMK2A levels were measured using ELISA. The plasma CaMK2A levels of the students with high and low cognitive ability were 529 and 271 pg/mL, respectively. Thus, plasma CaMK2A was positively correlated with the cognitive ability of Taiwanese senior high school students (Table 4).

**Table 4:** Plasma CaMK2A is positively correlated with cognitive ability

	HMAT group (*n* = 60)	LMAT group (*n* = 60)	*T*	*p*
CaMK2A Plasma level (pg/ml)	523.3 (189.5) (pg/ml)	272.1 (214.3) (pg/ml)	6.80	< 0.001

^*^Mean values (standard deviation)

HMAT, Higher Multiple Aptitude Test; LMAT, Lower Multiple Aptitude Test

^***^p < 0.001; *n* = 120, Student’s *t* test

### Relationship between identified SNVs and plasma CaMK2A levels

In correlating SNVs and CaMK2 levels with the cognitive ability of adolescents, we found that *GRIN3B* rs4807399 with the CT genotype was significantly associated with higher plasma CaMK2A levels and cognitive ability (Table 5). Additionally, *GRIN3B* rs10417824 with the A allele (AA, AT) was significantly associated with higher plasma CaMK2A levels and cognitive ability. Elevated plasma CaMK2A levels might not reflect an increase in activity in the CNS. We address this issue in the Section “Discussion”.

**Table 5:** Association of GRIN family genotypes with plasma CaMK2A levels and cognitive ability

		N	Mean	SD	F	P
*GRIN3A_rs3739722*						
CaMK2A Conc	CC	70	401.98	240.01	3.041	> 0.05
	CT	43	424.45	224.42		
	TT	7	190.62	223.68		
Cognitive ability	CC	70	45.76	42.57	4.891	0.009*
	CT	43	56.40	40.61		
	TT	7	5	2.45		
*GRIN3B_ rs2240154*						
CaMK2A Conc	CC	44	427.66	258.17	0.686	> 0.05
	CT	56	371.80	222.85		
	TT	20	404.31	234.75		
Cognitive ability	CC	44	61.68	40.35	5.219	0.007*
	CT	56	42.34	41.88		
	TT	2	28.90	37.58		
*GRIN3B_rs4807399*						
CaMK2A Conc	CC	98	374.67	239.60	4.299	0.016*
	CT	20	528.64	184.27		
	TT	2	216.90	220.70		
Cognitive ability	CC	98	42.75	41.58	3.377	0.037*
	CT	20	69.05	37.89		
	TT	2	46.50	64.35		
*GRIN3B_2240157*						
CaMK2A Conc	CC	42	429.98	262.68	0.679	> 0.05
	CT	55	373.07	221.50		
	TT	23	397.67	230.31		
Cognitive ability	CC	42	62.29	40.31	5.35	0.006*
	CT	55	43.02	41.93		
	TT	23	29.61	37.79		
*GRIN3B_rs2285906*						
CaMK2A Conc	AA	3	342.32	278.78	1.081	> 0.05
	AG	41	441.34	240.59		
	GG	76	376.35	234.66		
Cognitive ability	AA	3	35.33	49.23	4.032	0.020*
	AG	41	61.93	39.58		
	GG	76	39.71	41.55		
*GRIN3B_rs10417824*						
CaMK2A Conc	AA	18	457.39	270.92	4.686	0.011*
	AT	60	441.06	220.98		
	TT	42	310.19	226.06		
Cognitive ability	AA	18	70.94	35.94	8.571	0.000*
	AT	60	53.13	42.42		
	TT	42	28.52	36.80		
*GRIN3B_rs10401454*						
CaMK2A Conc	CC	75	371.39	223.99	1.489	> 0.05
	CG	35	454.98	259.31		
	GG	10	394.64	248.75		
Cognitive ability	CC	75	39.09	41.74	4.391	0.014*
	CG	35	57.51	40.78		
	GG	10	71.80	34.07		

^*^p value < 0.05

### CaMK2A upregulation triggers autophosphorylation signaling cascade

**Fig. 2:**
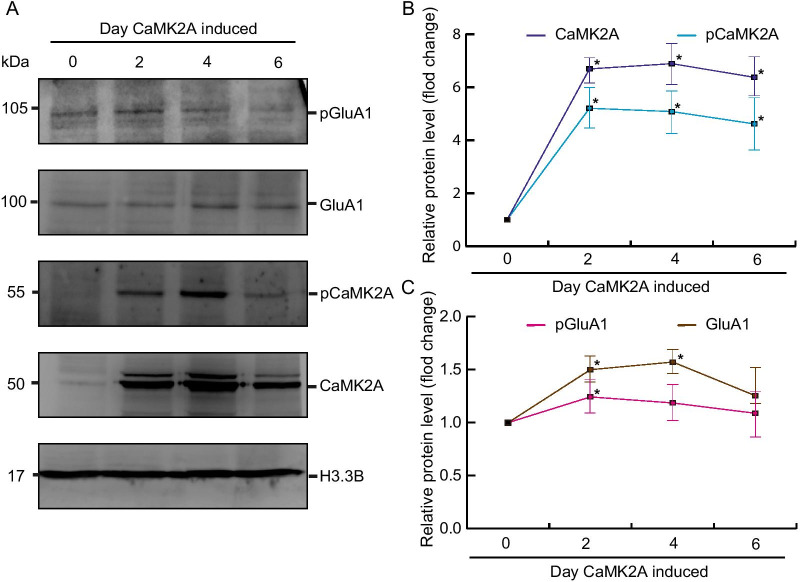
Increased CaMK2A activated autophosphorylation. Total soluble protein from HEK293-derived cells was harvested at 2, 4, and 6 days after induction of CaMK2A expression with doxycycline. **A** Representative immunoblots displaying CaMK2A, phospho-CaMK2A (T286), GluA1, and phospho-GluA1 (S831) expression. H3.3B was considered the loading control. **B**, **C** Autophosphorylation of CaMK2A and phosphorylation of GluA1 increased after CaMK2A induction. Quantification of relative protein levels. Data are presented as mean ± standard deviation. *p < 0.05, *n* = 5, Student’s *t* test

To assess the effect of CaMK2A upregulation in vivo, quantitative immunoblotting was performed using a human-derived neuroblastoma SH-SY5Y cell line because it has been used to study the relations of epigenetic biomarkers with academic performance and neuronal disorders associated with neurocognitive disorders [36, 46–50]. CaMK2A autophosphorylation increased proportionately with its expression, plateauing 2 days after CaMK2A upregulation CaMK2A expression persisted through day 6, whereas pCaMK2A had been slightly downregulated by day 6 (Fig. 2B). Additionally, GluAl, a component of the AMPAR, is phosphorylated by CaMK2 at S831. GluA1 was concomitantly upregulated with pCaMK2A upregulation on days 2–4 and had been downregulated by day 6. CaMK2 and GluA1 phosphorylation were both increased, suggesting that CaMK2A upregulation might initiate CaMK2 phosphorylation and phosphorylate downstream targets such as GluA1 (Fig. 2C).

### MeCP2 is involved in CaMK2A-mediated phosphorylation regulation

**Fig. 3:**
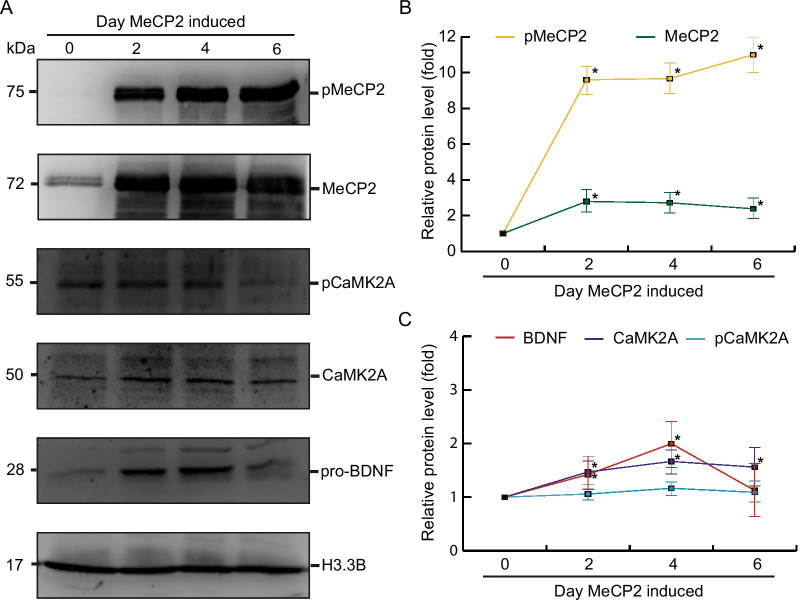
MeCP2 is involved in CaMK2A-mediated phosphorylation. Total soluble protein from stably transfected SH-SY5Y cells was harvested at 2, 4, and 6 days after induction of MeCP2 expression with doxycycline. **A** Representative immunoblots displaying phospho-MeCP2 (S80), MeCP2, phospho-CaMK2A (T286), CaMK2A, and pro-BDNF expression. H3.3B was considered the loading control. **B**, **C** Quantification of relative protein expression levels. Data are presented as mean ± standard deviation. *p < 0.05, *n* = 5, Student’s *t* test

We previously reported the involvement of a homeostatic regulatory control mechanism of MeCP2, similar to that of CaMKII, in both Rett syndrome and synaptic plasticity [46]. CaMKII autophosphorylation is essential for LTP induction and memory consolidation, and MeCP2 is a direct target of CaMKII; therefore, we investigated whether MeCP2 is involved in an autoregulatory loop that activates CaMKII and increases CaMKII autophosphorylation. Quantitative immunoblotting revealed that CaMK2A and phospho-MeCP2 (S80) were significantly upregulated 2 days after MeCP2 induction in stably transfected SH-SY5Y cells. The elevated expression persisted through day 6 after MeCP2 induction. This finding indicates that CaMK2A and MeCP2 may form a positive feedback loop. Furthermore, BDNF, a downstream target of MeCP2, was significantly upregulated upon MeCP2 overexpression in SH-SY5Y cells, indicating that phospho-MeCP2 was released by the BDNF promoter, thereby facilitating BDNF expression (Fig 3).

### *GRIA1* and *GRID1* promoter regions are direct targets of MeCP2

**Fig. 4:**
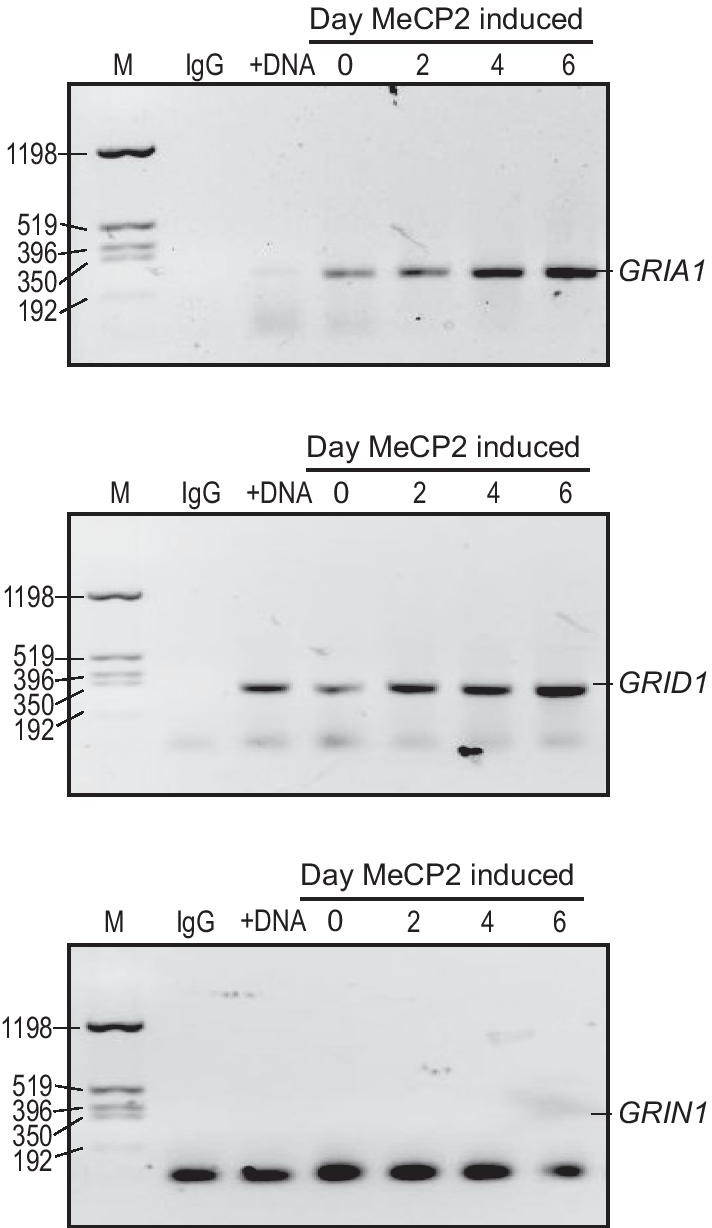
MeCP2 binds to *GRIA1* and *GRID1* promoters*.* ChIP was performed using anti-MeCP2 antibodies on sheared chromatin from SH-SY5Y neuroblastoma cells expressing MeCP2 on days 0, 2, 4, and 6. Purified DNA from immunoprecipitated chromatin was amplified using optimized primers for the promoters (*GRIA1*, *GRID1*, and *GRIN1*). Molecular weight markers are on the left in bases. Normal rabbit IgG was used as a negative immunoprecipitation control. +DNA: Purified chromosomal DNA, used as a positive control

To determine the correlation between the identified SNVs and their potential regulation of promoter binding regulation we performed ChIP to investigate whether MeCP2 binds to the promoter regions encompassed by these SNVs (e.g., *GRIA1* promoter 548294, *GRID1* promoter 3814614, and *GRIN1* promoter 4880213). An anti-MeCP2 antibody could pull down the promoters of *GRIA1* and *GRID1* in SH-SY5Y neurons, indicating that MeCP2 directly regulates *GRIA1* and *GRID1* (Fig. 4). By contrast, MeCP2 could not bind to the *GRIN1* promoter (Fig. 4). Furthermore, the binding of MeCP2 to the *GRIA1* and *GRID1* promoters was time-dependent, indicating that *GRIA1* and *GRID1* are direct downstream targets of MeCP2 (Fig. 4).

## Discussion

In this study, we investigated the correlations of the genetic variations and expression of genes involved in synaptic plasticity with the cognitive function of senior high school students. To identify genetic variants related to cognitive ability, we studied members of the NMDA-dependent AMPAR trafficking cascade, including *GRIN1*, *GRIN2B*, *GRIN2C*, *GRIN3A*, *GRIN3B*, *GRIA1*, and *CaMK2A*, because the functions of glutamate receptors and CaMKII are involved in neural plasticity and memory [51]. The repeated action-potential firing and subsequent calcium influx induce autonomous activation of CaMKII, which is essential for memory formation. The activation of CaMKII is also closely linked to the NMDAR, whose main function is synaptic restructuring and long-term memory formation [52, 53]. Plasma CaMK2A levels were positively associated with the cognitive ability of students. Using cultured cell systems, we discovered alterations in the levels of the aforementioned proteins, including MeCP2, pMeCP2, CaMK2A, pCaMK2A, GluA1, and pGluA1. We believe that using a simpler cultured cell system could aid in uncovering the role of these elementary building blocks of learning and memory in more complex organisms.

The students in our study were physically healthy and from homogeneous socioeconomic areas; therefore, our results may not apply more broadly to a heterogeneous population. Additionally, although our effective sample size was small, we detected the associations between SNVs in glutamatergic system genes and the cognitive ability of Taiwanese senior high school students. Specifically, variants of *CaMK2A* (rs2241694) and several glutamatergic system genes including *GRIA1*, *GRIN1*, *GRIN2*, *GRIN3A*, and *GRIN3B* were associated with cognitive ability. Of all 174 SNVs in our genetic association study, those of *GRIN2A* and *GRIN2D* were excluded because of their low minor allele frequency (< 5%). This finding suggests that the major alleles for the *GRIN2A* and *GRIN2D* SNVs might be conserved in the Han Chinese population. Furthermore, the *GRIN2C* rs3744215 SNV was significantly associated with abstract reasoning. In addition to the cerebellum, GluN2C expression was also detected in several first- and higher-order thalamic nuclei, vestibular nuclei, and parvalbumin-positive interneurons [54]. Lesions in the cerebellum, especially in the posterior lobes, can impair executive function, including planning, abstract reasoning, and working memory [55]. Moreover, a recent meta-analysis revealed that the subdivisions of the thalamus are associated with different cognitive functions [56]. These findings suggest that cerebellum-enriched GluN2C might function in abstract reasoning.

Consistent with our findings, several *GRIN3B* variants were previously associated significantly with cognitive function [57]. *GRIN3B*, an unconventional member of the NMDAR family, complexes with the *GluN1* and *GluN2* subunits and modulates Ca^2+^ permeability and membrane trafficking [58, 59]. However, stimulation of unconventional receptors, such as GluN3B, may compete against conventional NMDARs (GluN1/GluN2) for synaptic depotentiation in response to subsequent synaptic stimulation [60]. This metaplasticity is thought to be involved in LTP, long-term depression, and memory storage. Further research to examine how the *GRIN3B* variations affect the function of glutamatergic receptors and the cognitive ability of students is warranted.

Using ELISA, we discovered that students who performed well on the MAT had higher plasma CaMK2A, whereas students who scored poorly on the MAT had lower plasma CaMK2A. CaMK2A concentrations quantified through mass spectrometry and ELISA are compatible because both are in picogram per liter ranges (https://www.proteinatlas.org/ENSG00000070808-CAMK2A/blood). CaMK2A is expressed mostly in the CNS, adrenal gland, stomach, kidneys, liver, and bone marrow (https://www.proteinatlas.org/ENSG00000070808-CAMK2A/tissue). It can also be detected in neutrophils and memory B cells in the circulatory system. Although we do not know the source of plasma CaMK2A or how it is secreted into serum, ELISA is sufficiently sensitive for measuring it in blood samples. Because blood samples are more accessible than neuronal tissue samples, plasma proteins and peptides exported from the brain in blood samples would be ideal for practical exploration of the biomarkers that affect cognitive ability in young people.

The expression of pCaMK2A (Thr286), GluA1, and pGluA1 (Ser831) were increased was elevated in the stably transfected HEK293 cells expressing CaMK2A, suggesting that CaMK2A could initiate an autophosphorylation cascade, thereby activating the downstream target genes (Fig. 2). Additionally, MeCP2, a downstream target of CaMK2A, and BDNF can be activated by MeCP2 in SH-SY5Y cells (Fig. 3). Although we are unsure whether MeCP2, a transcription regulator that binds to methylated DNA, activates CaMK2A directly, our result demonstrates that CaMK2A and MeCP2 could form an autoregulatory loop. Notably, a 1.5-fold increase occurred in CaMK2A, but pCaMK2A was not increased in SH-SY5Y cells expressing MeCP2 (Fig. 3). The CaMK2A increase may be insufficient to initiate its autophosphorylation. However, a Ca^2+^ influx may be initially required to trigger CaMK2A phosphorylation, and that cytosolic Ca^2+^ might have been insufficient to initiate CaMK2A autophosphorylation in MeCP2-expressing cells. We believe that a sufficient amount of the upregulated CaMK2A may be autophosphorylated when a Ca^2+^ influx is triggered by neuronal activity.

**Fig. 5:**
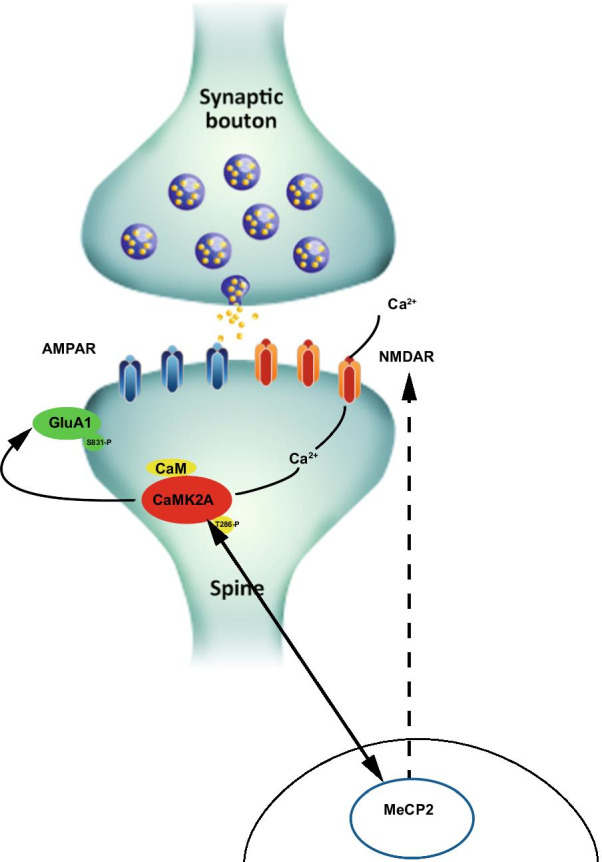
MeCP2 interaction with CaMK2A and CaMK2A phosphorylation signaling pathway plays a critical role in synaptogenesis and molecular mechanism of learning

In summary, we found that SNVs in *CaMK2A* and subunits of ionotropic glutamate receptors, including *GRIA1*, *GRIN1*, *GRIN2*, and *GRIN3*, were associated with the cognitive function of students (Tables 1,2,3). The levels of CaMK2A were elevated in the peripheral blood samples of senior high school students with strong reasoning skills, especially in those with *GRIN3B* variants. These results indicate that the primary function of the CaMK2A phosphorylation signaling pathway is critical in synaptogenesis and the molecular mechanism of learning (Fig. 5). The physiological and pathological roles of CaMK2A have attracted substantial attention due to their involvement in synaptic plasticity. Our SH-SY5Y-derived CaMK2A model is an appropriate system to study the associations of synaptogenesis, LTP, and long-term depression and the cognitive function of students.

## Limitations

Although the students were of similar ages and had similar educational backgrounds, the male students outperformed the female students in basic competency test (BCT), numerical ability, mechanical reasoning, spatial relations, and abstract reasoning, whereas female students performed better in verbal comprehension and grammar and language usage (Table 1). We cannot attribute the differences in cognition to sex alone, because human cognition is highly complex and may be influenced by the environment, culture, and individual experience [61]. Moreover, because we could not assess these factors quantitatively, over-interpretation of results and speculation regarding the cause of the results would be inappropriate.

We found that plasma CaMK2A levels were correlated with the cognitive ability of students (Table 4). To further assess the consequences of CaMK2A upregulation in vivo, we quantified the expression levels of several components of glutamatergic signaling in a human-derived SH-SY5Y cell line (Figs. 2 and 3). Although the cultured neuroblastoma cells enabled quick assessment, noninvasive monitoring of gene expression in the brain would be more informative.

## Data Availability

The data and materials that support the findings of this study are available from the corresponding author upon reasonable request.

## Abbreviations

ANOVA: Analysis of variance
AMPAR: α-Amino-3-hydroxy-5-methyl-4-isoxazolepropionic acid receptor
BCT: Basic competency test
BDNF: Brain-derived neurotrophic factor
Cam: Calmodulin
CaMK2A: Calcium/calmodulin-dependent protein kinase IIα
CNS: Central nervous system
EDTA: Ethylenediaminetetraacetic acid
ELISA: Enzyme-linked immunosorbent assay
*GRINs*: Glutamate ionotropic receptor N-methyl-D-aspartate type subunits genes (*GRIN1*, *GRIN2A-D*, *GRIN3A,3B*)
GluA1: Glutamate ionotropic receptor α-amino-3-hydroxy-5-methyl-4-isoxazolepropionic acid type subunit 1
GluD1: Glutamate ionotropic receptor delta type subunit 1
GluN1: Glutamate ionotropic receptor type subunit 1
GluN2B: Glutamate ionotropic receptor N-methyl-D-aspartate type subunit 2B
GluN2C: Glutamate ionotropic receptor N-methyl-D-aspartate type subunit 2C
GluN3A: Glutamate ionotropic receptor N-methyl-D-aspartate type subunit 3A
GluN3B: Glutamate ionotropic receptor N-methyl-D-aspartate type subunit 3B
LTP: Long-term potentiation
MAT: Multiple Aptitude Test Battery
MeCP2: Methyl-CpG binding protein 2
NMDAR: N-methyl-D-aspartate receptor
SNV: Single-nucleotide variant
